# Supraventricular tachycardia and urticaria complicating leuprolide-induced ovarian suppression in a young woman with breast cancer: a case report

**DOI:** 10.3332/ecancer.2013.335

**Published:** 2013-07-24

**Authors:** Sharan Prakash Sharma, Franco Muggia

**Affiliations:** 1 Department of Internal Medicine, Englewood Hospital and Medical Center, Englewood, 07631 NJ, USA; 2 Division of Medical Oncology, Department of Medicine, New York University School of Medicine, 10016 NY, USA

**Keywords:** breast cancer, leuprolide, supraventricular tachycardia, urticaria, gelatin

## Abstract

Gonadotropin-releasing hormone (GnRH) agonists are used for gonadal suppression in the treatment of breast and prostate cancers. In older men, their use has occasionally been associated with cardiovascular side effects such as supraventricular tachyarrhythmias (SVTs). Several reports document their occurrence in men receiving leuprolide for prostate cancer. We now report this complication with concomitant occurrence of migratory trunk and extremity urticaria in a young woman receiving this treatment after diagnosis of a T1cN0 premenopausal breast cancer. Changing from leuprolide to another GnRH agonist, goserelin, no additional problems with SVT or accompanying urticaria were encountered during the nearly two years of treatment and three subsequent years of follow-up.

## Introduction

Gonadotropin-releasing hormone (GnRH) agonists are decapeptides that exert a nonpulsatile constant stimulation to the anterior pituitary gland, which, after an initial transient increase in LH, causes downregulation of gonadotropin release resulting in inhibition of sex hormone production [1].

GnRH analogue formulated in depot forms, such as leuprolide and goserelin, have been widely used in monthly or trimonthly administration for a variety of diseases that may benefit from gonadal suppression. These include prostate cancer, breast cancer, endometriosis, uterine leiomyomas and central precocious puberty [2]. Many adverse reactions have been reported with leuprolide from local skin manifestations ranging from rash to urticarial and severe anaphylactoid reactions [3, 4]. Similarly, there have been reports from systemic hypersensitivity to recurrent anaphylaxis with goserelin [5, 6].

Studies have also shown an increased risk to manifest metabolic syndrome, diabetes mellitus and cardiovascular disease in men receiving GnRH analogues for prostate cancer [7]. Adverse effects caused by GnRH analogue administration have been attributed to the reduced levels of circulating testosterone [8, 9]. As a result, men receiving GnRH agonists are associated with a greater risk of myocardial infarction and sudden cardiac death [10]. In addition, GNRH agonists can also cause prolonged QT syndrome [11].

A few studies have reported the occurrence of cardiac arrhythmia in leuprolide users. In a randomised open-label trial comparing leuprolide with a GnRH antagonist, degarelix in prostate cancer found that the most common type of arrhythmia was supraventricular (SVT), occurring in 2% of patients in the degarelix group and 4% in the leuprolide group [12].

It is hypothesised that a GnRH agonist could regulate cardiac contractility and intracellular calcium ion concentration via a GnRH receptor/protein kinase A (PKA)-dependent mechanism [13]. PKA has been implicated in leuprolide-associated SVT.

Based on the data from the US Food and Drug Administration, an analysis conducted by a medical analysis website company revealed that of 4,487 people who reported side effects on Lupron 0.57% experienced SVTs. Almost 90% of them were male and over 60 years of age [14]. We have found no studies that have looked into the occurrence of arrhythmia in breast cancer of women taking GnRH agonists. In addition, the concomitant occurrence of migratory urticaria is noteworthy.

## Case presentation

A 40-year-old Caucasian lawyer in excellent health except for migraines was diagnosed with 2-cm right breast cancer moderately differentiated and estrogen and progesterone receptors strongly positive, and negative for Her2 and low Ki67. She underwent a lumpectomy with negative axillary sentinel node biopsy. Her oncotype DX score was 12; with this low score, she was started on tamoxifen with Lupron-induced ovarian suppression. After eight months of monthly Lupron therapy, she started to have SVT together with the appearance of an urticarial rash. Two episodes of SVT required emergency hospital visits; a Holter monitoring for 24 h documented additional episodes, but they each lasted less than 5 minutes in duration. Her treatment was changed to goserelin on the premise that the troublesome urticaria represented an allergic reaction. Shortly after this change, both urticarial and episodes of SVT abated; she remained symptoms-free until completion of her adjuvant hormonal treatment at 36 months. Her haemoglobin, chemistry and liver function remained normal throughout the treatment. Her echocardiography result was also normal. As of her last examination in March 2013, she remains disease-free, five years after diagnosis; her menstrual periods resumed a few months after cessation of goserelin and tamoxifen. Additional years of endocrine therapy were abrogated because pregnancy was being considered. After leuprolide was stopped, the patient completed three years of combined treatment with tamoxifen and goserelin without any problems.

## Discussion

Studies have occasionally reported cross reactivity between different types of GnRH agonists causing allergic reactions [15] when one GnRH agonist was switched to another. However, our case did not demonstrate the recurrence of urticaria or SVT tachycardia when leuprolide was changed to goserelin. Their concomitant occurrence points to the possibility of some allergic components in the development of the SVT. A comparison of the vehicles suggests that while leuprolide is formulated in gelatin, goserelin has other stabilisers. Gelatin-containing vaccines such as MMR, varicella, and DTaP have been shown to cause allergic reactions ranging from localised erythaema to severe urticaria and systemic reactions [16, 17]. However, SVT has not been reported in association with allergy to gelatin. This case report further highlights the possibility of SVT occurring not only in older men being treated for prostate cancer but also in young patients with breast cancer following leuprolide.

Our patient’s course implies that allergy to gelatin could be the inciting factor behind the development of urticaria and arrhythmia. The management of SVT includes adenosine, a very short-acting endogenous nucleotide that blocks atrioventricular nodal conduction and terminates nearly all atrioventricular nodal reentrant tachycardias and atrioventricular reciprocating tachycardias, but in this instance, such a manoeuvre was not necessary [18].

## Conclusion

Young women receiving leuprolide may be at risk for SVT, and it should be considered in the differential diagnosis of palpitation/chest pain. The relationship between urticaria and the arrhythmia remains obscure.
